# Associations between specialized dementia care, COVID-19 and central nervous system medication use in assisted living: a population-based repeated cross-sectional study

**DOI:** 10.1186/s12877-024-05274-w

**Published:** 2024-08-14

**Authors:** Colleen J. Maxwell, Hana Dampf, Jillian P. Squires, David B. Hogan, Cecilia A. Cotton, Erik Youngson MMath, Zoe Hsu, Matthias Hoben

**Affiliations:** 1 School of Pharmacy, University of Waterloo, 200 University Avenue West, Waterloo, ON N2L 3G1 Canada; 2 ICES, 2075 Bayview Avenue, Toronto, ON V1 06, M4N 3M5 Canada; 3 Faculty of Nursing, College of Health Sciences, University of Alberta, Edmonton, AB T6G 2R3 Canada; 4 Division of Geriatric Medicine, Cumming School of Medicine, University of Calgary, 3330 Hospital Drive NW, Calgary, AB T2N 4N1 Canada; 5 Department of Statistics and Actuarial Science, University of Waterloo, 200 University Avenue West, Waterloo, ON N2L 3G1 Canada; 6 Provincial Research Data Services, Alberta Health Services, Edmonton, AB T6G 2C8 Canada; 7 Data and Research Services, Alberta SPOR SUPPORT Unit, Edmonton, AB T6G 2C8 Canada; 8 School of Health Policy and Management, Faculty of Health, York University, Toronto, ON M3J 1P3 Canada

**Keywords:** Assisted living, Dementia care unit, Psychotropic, Anticonvulsant, Anti-dementia therapy, COVID-19

## Abstract

**Background:**

Assisted living (AL) is an increasingly common residential setting for persons with dementia; yet concerns exist about sub-optimal care of this population in AL given its lower levels of staffing and services. Our objectives were to (i) examine associations between AL setting (dementia care vs. other), COVID-19 pandemic waves, and prevalent antipsychotic, antidepressant, anti-dementia, benzodiazepine, and anticonvulsant drug use among residents with dementia/cognitive impairment, and (ii) explore associations between resident and home characteristics and prevalent medication use.

**Methods:**

We conducted a population-based, repeated cross-sectional study using linked clinical and health administrative databases for all publicly funded AL homes in Alberta, Canada, examined between January 2018 - December 2021. The quarterly proportion of residents dispensed a study medication was examined for each setting and period (pandemic vs. comparable historical [2018/2019 combined]) focusing on four pandemic waves (March-May 2020, September 2020-February 2021, March-May 2021, September-December 2021). Log-binomial GEE models estimated prevalence ratios (PR) for period (pandemic vs. historical periods), setting (dementia care vs. other) and period-setting interactions, adjusting for resident (age, sex) and home (COVID-19 cases, health region, ownership) characteristics.

**Results:**

On March 1, 2020, there were 2,779 dementia care and 3,013 other AL residents (mean age 83, 69% female) with dementia/cognitive impairment. Antipsychotic use increased during waves 2–4 in both settings, but this was more pronounced in dementia care than other AL during waves 3 and 4 (e.g., adjusted [adj]PR 1.20, 95% CI 1.14–1.27 vs. adjPR 1.09, 95% CI 1.02–1.17, interaction *p* = 0.023, wave 3). Both settings showed a statistically significant but modest increase in antidepressant use and decrease in benzodiazepine use. For dementia care AL residents only, there was a statistically significant increase in gabapentinoid use during several waves (e.g., adjPR 1.32, 95% CI 1.10–1.59, wave 3). Other than a modest decrease in prevalent anti-dementia drug use for both settings in wave 2, no other significant pandemic effects were observed.

**Conclusions:**

The persistence of the pandemic-associated increase in antipsychotic and antidepressant use in AL residents coupled with a greater increase in antipsychotic and gabapentinoid use for dementia care settings raises concerns about the attendant risks for residents with cognitive impairment.

**Supplementary Information:**

The online version contains supplementary material available at 10.1186/s12877-024-05274-w.

## Background

More than half of individuals living in assisted living (AL) settings have dementia [1, 2], yet there are questions regarding the capacity of these settings to care for this population [3, 4]. AL aims to promote independence and quality of life in a home-like setting. Relative to nursing homes, AL settings have lower staffing and service levels with no or limited access to professional nurses on site [1, 3]. A systematic review comparing nursing homes (NHs) and other residential care settings including AL found that residents with mild dementia in NHs had fewer hospitalizations and more stable health, likely explained by the higher level of health care and staffing provided by NHs [5]. The impact of the COVID-19 pandemic on staffing and services across diverse congregate care settings may have exacerbated these differences in resident care and outcomes [6, 7].

Individuals with dementia present unique care challenges, including the need for more assistance with activities of daily living (ADLs) and a higher prevalence of both responsive behaviours and depression [8]. Persons with dementia living in AL frequently have frailty, multiple comorbidities and hyperpolypharmacy [1, 2, 4, 9], placing them at greater risk of adverse outcomes such as falls and hospitalizations [10]. It is plausible that the COVID-19 pandemic and related consequences of infection control measures, including resident isolation and lack of family involvement, exacerbated dementia-related behavioural symptoms [11]. Among other factors, this increase in responsive behaviours along with the reduced availability of staff with adequate training and disruption of non-pharmacological behavioural interventions, may have led to an increased use of medications that act on the central nervous system (CNS) [12].

The adverse effects and uncertain benefits associated with antipsychotics and other select CNS medications are well documented [13–15], yet these agents are commonly used to manage dementia-associated behavioural symptoms [14, 16, 17]. There was increased use of psychotropic and other CNS medications among nursing home residents during the first two waves of the COVID-19 pandemic [12, 18–21]. Fewer studies have explored whether comparable CNS medication changes occurred among AL residents, despite the growing importance of AL settings in providing care for persons with dementia and their unique features relative to nursing homes. A recent investigation of publicly subsidized AL homes in Alberta, Canada showed a significant increase in antipsychotic and antidepressant use during COVID-19 [22]. The increase in antipsychotic use was significantly greater for residents of AL sites designated as providing dementia care than other AL homes. However, this earlier study did not comprehensively examine COVID-19 associated CNS medication trends among a more cognitively vulnerable AL cohort, including the use of anti-dementia drugs (cholinesterase inhibitors, memantine), or explore the relevance of residents’ age and sex and AL home characteristics (including resident COVID-19 cases), in adjusted models [22].

The primary objective of the current study was to examine variations in prevalent antipsychotic, antidepressant, anti-dementia, benzodiazepine, gabapentinoid and other anticonvulsant drug use (CNS drugs) among AL residents with dementia and/or moderate or greater cognitive impairment, across COVID-19 pandemic waves 1–4 (relative to corresponding historical periods) and by setting (designated dementia care vs. other AL). Our secondary objective was to explore associations between select AL resident (age, sex) and home (presence of COVID-19 among residents, ownership status and health region location) characteristics and prevalent CNS drug use throughout this COVID-19 period. We hypothesized that COVID-19 would be associated with changes in prevalent CNS drug use in this cognitively vulnerable AL cohort and that some associations would differ for those residing in designated dementia care vs. other AL settings. We further hypothesized that residents’ age and sex, but not home characteristics, would be significantly associated with prevalent CNS drug use, but that adjustment for these covariates would not meaningfully alter setting or period associations.

## Methods

### Study design and setting

We utilized the province of Alberta’s population-based clinical and health administrative databases and a repeated cross-sectional design to examine prevalent CNS medication use among cognitively vulnerable residents of publicly subsidized AL between January 2018 and December 2021. The cohort was restricted to residents with a dementia diagnosis and/or a moderate or greater cognitive impairment severity (i.e., Cognitive Performance Scale [CPS] score of 3+) (hereafter referred to as cognitive impairment). Publicly funded AL (termed designated supportive living [DSL]) in the province is overseen by Alberta Health Services (AHS) [23] and includes three levels of care: *DSL3* is suited for more stable and functionally independent residents, with care aides available 24/h a day; *DSL4* has care aides and a licenced practical nurse available 24/h a day and is suited for more complex residents requiring assistance with daily activities and/or mobility; and *DSL4D* is comparable to *DSL4*, but specifically designed to optimize safety for residents with moderate to severe dementia [23]. We stratified settings as dementia care (DSL4D) vs. other (DSL3/4) AL.

### Data sources and participants

Databases included the Discharge Abstract Database (DAD) for inpatient hospital stays, the Pharmaceutical Information Network database (PIN) for outpatient prescription medications (including those for AL residents), Vital Statistics for death dates, Provincial Laboratory for COVID-19 testing data (month/year only), Immunization & Adverse Reactions to Immunization (ImmARI) for aggregate data on vaccine administration, and the Alberta Continuing Care Information System (ACCIS) for data on continuing care residents (e.g., admissions/discharge dates, the Resident Assessment Instrument-Home Care [RAI-HC] assessment) and AL homes (see Additional File 1, Table S1). The RAI-HC is a standardized assessment of AL residents, administered on admission and at least annually, for the purpose of informing resident-centered care plans [24]. Items assessed with the tool form various outcome scales to quantify residents’ clinical complexity and functional status [25]. Databases were extracted from the AHS Enterprise Data Warehouse and provided by AHS as individual level de-identified data and linked using unique resident identifiers.

All AL residents with a RAI-HC assessment completed between January 1, 2018 and December 31, 2021 (plus any assessments completed during a 1-year look back) and meeting the eligibility criteria (i.e., with a dementia diagnosis on the RAI-HC and/or cognitive impairment indicated by a CPS score of 3+) were captured. The pandemic period was from March 1, 2020 to December 31, 2021, and the historical period from January 1, 2018 to February 29, 2020. At each monthly index date (first day of each month), residents alive and in an AL setting (dementia care or other) were eligible for the study and those with an overlapping hospitalization were excluded. On each index date we also accounted for any change in resident setting (e.g., transfer to NH or different AL, death).

### Measures

#### Exposures

The main exposures were time-period (COVID-19 pandemic quarter starting March-May 2020 compared to corresponding historical [2018/2019 combined] quarter) and setting (dementia care AL vs. other AL). Monthly resident cohorts were combined to capture quarterly periods for modeling (each 3 months, except for the last quarter which was 4 months to accommodate study end date). This permitted an examination of specific quarters representing the four COVID-19 pandemic waves in Alberta [26] (wave 1 [March-May 2020], 2 [September-November 2020 & December 2020-February 2021], 3 [March-May 2021], and 4 [September-December 2021]) with corresponding 2018/2019 historical periods.

#### Outcomes

Prevalent use of antipsychotic, antidepressant, anti-dementia, benzodiazepine, gabapentinoid, and other anticonvulsant medications was defined using the PIN database (January 2017-December 2021) (see Additional File 1, Table S2). First, Anatomical Therapeutic Chemical (ATC) codes, drug dispense date and days’ supply were utilized to define class-specific monthly prevalence (denominator equaled mid-month population). Prevalent users were defined as residents who were either dispensed or had a continuous supply of a study class medication after the start of each monthly index date and before the end of the month or leaving the monthly cohort. A maximum supply of 100 days was used with values above this rounded to 100. Second, to derive the quarterly proportion of residents dispensed each medication class, relevant monthly prevalence estimates (e.g., March-May, June-August, etc.) were averaged, weighted by population.

#### AL resident and home characteristics

The most recent RAI-HC assessment before each index date was used to obtain resident characteristics. These included resident’s age, sex, scores on validated RAI-HC outcome scales (i.e., the Activities of Daily Living Self-Performance Hierarchy Scale [ADL] [27], Cognitive Performance Scale [CPS] [28], Depression Rating Scale [DRS] [29], Changes in Health, End-Stage Disease, Signs and Symptoms Scale [CHESS; gauges health instability] [30], and Pain Scale) [31], behaviours, chronic health conditions, and frailty. A validated frailty index (FI), derived as the proportion of accumulated to potential health deficits based on 72 RAI-HC items, was used to categorize residents as robust (FI < 0.20), pre-frail (FI 0.2–0.3), or frail (FI > 0.3) [32].

The AL home’s postal code, AHS continuing care registries, and ACCIS database were used to characterize each AL home with respect to geographic health zone (5 different zones with oversight of healthcare decisions and service delivery), urban/rural location, bed size, and for-profit vs. non-for-profit ownership status. For resident COVID-19 cases, the variable was coded as 1 if the AL home had one or more positive COVID-19 test results in the index month or in the month preceding the index month (and coded 0 for no positive COVID-19 tests results in index or preceding month).

### Analysis

Descriptive analyses compared yearly (on March 1st ) resident characteristics for those in dementia care vs. other AL settings, with meaningful differences based on standardized differences of greater than 0.10 [33]. For each setting, the quarterly prevalence of each medication class was plotted for historical (2018/2019 combined) and pandemic periods.

For each quarter (starting March-May 2020), separate log-binomial generalized estimating equations (GEE) models were used to estimate prevalence ratios (PRs) for period (pandemic vs. historical), setting (dementia care vs. other AL) and period-setting interactions. Because of a known bias when using an exchangeable working correlation structure with time-varying covariates [34], the GEE models employed an independence correlation structure and a robust sandwich variance estimator to account for within subject correlation [35, 36]. Clustering by AL home was small (intracluster correlation coefficients between 0.01 and 0.11 for medication classes) and therefore not accounted for in the models. The above models were further adjusted for resident age, sex, COVID-19 positive cases and AL health zone and ownership status (home characteristics were modeled as resident-level variables, i.e., home-level exposure assigned to each resident in the home). We did not adjust for AL home urban/rural location or bed size as these variables were strongly associated with AL health zone location. All analyses were conducted with SAS 9.4 (SAS Institute Inc.).

We conducted two sensitivity analyses where we further adjusted our models for (1) resident cognitive impairment (i.e., CPS score) and separately, for (2) resident frailty level (i.e., FI score).

This study received ethics clearance from the University of Alberta Health Research Ethics Board (Pro00116520), University of Calgary Conjoint Health Research Ethics Board (pSite-22-0001), York University Office of Research Ethics-Human Participants Review Sub-Committee (e2022-239) and operation approval from AHS. As this study involved secondary analyses of existing de-identified health administrative databases, obtaining resident consent was not possible or required. As such, the need for informed consent was waived by the University of Alberta Health Research Ethics Board (Pro00116520), University of Calgary Conjoint Health Research Ethics Board (pSite-22-0001), and the York University Office of Research Ethics-Human Participants Review Sub-Committee (e2022-239).

## Results

During the study period, there were 129,294 dementia care AL and 138,939 other AL monthly records included after exclusions for deaths or hospitalizations at the time of monthly index dates (1.9% of records excluded), representing 6079 unique dementia care and 7348 other AL residents, with 704 unique residents seen in both setting types. We included data from all 250 provincial AL homes with residents meeting our eligibility criteria, nine homes (3.6%) included only dementia care, 133 (53.2%) included only other AL care, and 108 (43.2%) included care settings of both types.

On March 1, 2020, there were 2,779 dementia care and 3,013 other AL residents (mean age 83 years, 69% female) with a dementia diagnosis and/or cognitive impairment. Relative to residents in other AL settings, those in dementia care were more likely to be aged 75–84 (than 85 + years), to have higher levels of ADL and cognitive impairment, depressive symptoms, frailty, responsive behaviours, dementia, delusions, and hallucinations (Table 1). They were less likely to be assessed as having daily pain or other chronic conditions. The two settings were comparable in resident sex and health instability (CHESS score). Resident characteristics were relatively stable over time within each setting except for a decrease in the proportion with meaningful depressive symptoms.

**Table 1:** Yearly characteristics of AL residents with dementia and/or cognitive impairment, by setting type

Characteristic	March 2019		March 2020		March 2021		Dec 2021	
	Dementia Care (n = 2641)	Other AL (n = 2899)	Dementia Care (n = 2779)	Other AL (n = 3013)	Dementia Care (n = 2711)	Other AL (n = 2851)	Dementia Care (n = 2817)	Other AL (n = 2931)
Age, mean (SD), y	82.7 (9.5)	83.5 (9.9)	82.8 (9.3)	83.4 (10.1)	82.9 (9.3)	83.4 (10.4)	82.7 (9.3)	83.2 (10.5)
Age group, y								
< 65	137 (5.2)	151 (5.2)	127 (4.6)	158 (5.2)	127 (4.7)	174 (6.1)	143 (5.1)	171 (5.8)
65–74	268 (10.1)	307 (10.6)	307 (11.0)	329 (10.9)	287 (10.6)	314 (11.0)	306 (10.9)	328 (11.2)
75–84	936 (35.4)^a^	848 (29.3)	987 (35.5)^a^	906 (30.1)	969 (35.7)^a^	822 (28.8)	1013 (36.0)^a^	880 (30.0)
85+	1300 (49.2)^a^	1593 (54.9)	1358 (48.9)	1620 (53.8)	1328 (49.0)^a^	1541 (54.1)	1355 (48.1)	1552 (53.0)
Sex								
Female	1801 (68.2)	2007 (69.2)	1912 (68.8)	2084 (69.2)	1899 (70.1)	1975 (69.3)	1999 (71.0)	1970 (67.2)
Male	840 (31.8)	892 (30.8)	867 (31.2)	929 (30.8)	812 (29.5)	876 (30.7)	818 (29.0)	961 (32.8)
ADL Performance (score)								
Independent/Supervised (0,1)	1114 (42.2)^a^	1557 (53.7)	1213 (43.6)^a^	1614 (53.6)	1131 (41.7)^a^	1485 (52.1)	1154 (41.0)^a^	1523 (52.0)
Limited assistance (2)	697 (26.4)	648 (22.4)	715 (25.7)^a^	640 (21.2)	706 (26.0)	674 (23.6)	762 (27.0)	683 (23.3)
Extensive assistance/Dependent (3+)	830 (31.4)^a^	694 (23.9)	851 (30.6)^a^	759 (25.2)	874 (32.2)^a^	692 (24.3)	901 (32.0)^a^	725 (24.7)
Cognitive Performance Scale (score)								
Intact/Borderline Intact (0,1)	48 (1.8)^a^	405 (14.0)	59 (2.1)^a^	432 (14.3)	57 (2.1)^a^	391 (13.7)	58 (2.1)^a^	381 (13.0)
Mild impairment (2)	800 (30.3)^a^	1640 (56.6)	859 (30.9)^a^	1771 (58.8)	891 (32.9)^a^	1714 (60.1)	904 (32.1)^a^	1741 (59.4)
Moderate to severe impairment (3+)	1793 (67.9)^a^	854 (29.5)	1861 (67.0)^a^	810 (26.9)	1763 (65.0)^a^	746 (26.2)	1855 (65.9)^a^	809 (27.6)
Depression Rating Scale (score)								
No meaningful depressive symptoms (0–2)	1951 (73.9)^a^	2373 (81.9)	2091 (75.2)^a^	2533 (84.1)	2098 (77.4)^a^	2439 (85.5)	2217 (78.7)^ab^	2539 (86.6)^b^
Meaningful depressive symptoms (3+)	690 (26.1)^a^	526 (18.1)	688 (24.8)^a^	480 (15.9)	613 (22.6)^a^	412 (14.5)	600 (21.3)^ab^	392 (13.4)^b^
CHESS Scale (score)								
No health instability (0)	1764 (66.8)^a^	1773 (61.2)	1869 (67.3)	1897 (63.0)	1803 (66.5)	1842 (64.6)	1873 (66.5)	1874 (63.9)
Some health instability (1)	560 (21.2)	720 (24.8)	580 (20.9)	740 (24.6)	619 (22.8)	670 (23.5)	616 (21.9)	690 (23.5)
Higher health instability (2+)	317 (12.0)	406 (14.0)	330 (11.9)	376 (12.5)	289 (10.7)	339 (11.9)	328 (11.6)	367 (12.5)
Frailty (FI value)								
Robust (< 0.2)	547 (20.7)^a^	842 (29.0)	563 (20.3)^a^	924 (30.7)	545 (20.1)^a^	838 (29.4)	584 (20.7)^a^	882 (30.1)
Prefrail (0.2–0.3)	1075 (40.7)	1130 (39.0)	1181 (42.5)^a^	1123 (37.3)	1130 (41.7)	1069 (37.5)	1143 (40.6)	1110 (37.9)
Frail (> 0.3)	1019 (38.6)^a^	927 (32.0)	1035 (37.2)^a^	966 (32.1)	1036 (38.2)^a^	944 (33.1)	1090 (38.7)^a^	939 (32.0)
# Responsive behaviours^c^								
0	1456 (55.1)^a^	2268 (78.2)	1584 (57.0)^a^	2352 (78.1)	1581 (58.3)^a^	2221 (77.9)	1675 (59.5)^a^	2299 (78.4)
1	648 (24.5)^a^	428 (14.8)	680 (24.5)^a^	473 (15.7)	641 (23.6)^a^	467 (16.4)	668 (23.7)^a^	448 (15.3)
2+	537 (20.3)^a^	203 (7.0)	515 (18.5)^a^	188 (6.2)	489 (18.0)^a^	163 (5.7)	474 (16.8)^a^	184 (6.3)
Pain Scale (score)								
No pain (0)	1768 (66.9)^a^	1527 (52.7)	1854 (66.7)^a^	1630 (54.1)	1867 (68.9)^a^	1581 (55.5)	1950 (69.2)^a^	1659 (56.6)
Less than daily pain (1)	352 (13.3)	416 (14.3)	378 (13.6)	437 (14.5)	373 (13.8)	451 (15.8)	378 (13.4)	452 (15.4)
Mild/Moderate daily pain (2)	481 (18.2)^a^	849 (29.3)	503 (18.1)^a^	850 (28.2)	446 (16.4)^a^	738 (25.9)	465 (16.5)^a^	735 (25.1)
Severe daily pain (3)	40 (1.5)^a^	107 (3.7)	44 (1.6)^a^	96 (3.2)	25 (0.9)^a^	81 (2.8)	24 (0.9)^a^	85 (2.9)
Chronic Conditions, mean (SD)	3.69 (1.69)	4.34 (1.87)	3.73 (1.66)	4.37 (1.86)	3.75 (1.66)	4.40 (1.90)	3.75 (1.68)	4.38 (1.90)
# Chronic Conditions								
0–2	682 (25.8)^a^	449 (15.5)	681 (24.5)^a^	446 (14.8)	645 (23.8)^a^	418 (14.7)	673 (23.9)^a^	453 (15.5)
3,4	1188 (45.0)	1189 (41.0)	1257 (45.2)	1250 (41.5)	1249 (46.1)	1181 (41.4)	1279 (45.4)^a^	1178 (40.2)
5+	771 (29.2)^a^	1261 (43.5)	841 (30.3)^a^	1317 (43.7)	817 (30.1)^a^	1252 (43.9)	865 (30.7)^a^	1300 (44.4)
Selected Health Conditions								
Dementia	2555 (96.7)^a^	2676 (92.3)	2687 (96.7)^a^	2813 (93.4)	2629 (97.0)^a^	2647 (92.8)	2736 (97.1)^a^	2702 (92.2)
Arthritis	1082 (41.0)^a^	1434 (49.5)	1174 (42.2)^a^	1491 (49.5)	1176 (43.4)^a^	1443 (50.6)	1207 (42.8)^a^	1454 (49.6)
Diabetes	482 (18.3)^a^	691 (23.8)	482 (17.3)^a^	725 (24.1)	458 (16.9)^a^	693 (24.3)	468 (16.6)^a^	727 (24.8)
Chronic Obstructive Pulmonary Disease	418 (15.8)^a^	626 (21.6)	446 (16.0)^a^	630 (20.9)	402 (14.8)^a^	577 (20.2)	417 (14.8)^a^	588 (20.1)
Congestive Heart Failure	174 (6.6)^a^	375 (12.9)	163 (5.9)^a^	403 (13.4)	149 (5.5)^a^	380 (13.3)	153 (5.2)^a^	881 (13.7)
Cancer	158 (6.0)	175 (6.0)	152 (5.5)	183 (6.1)	150 (5.5)	176 (6.2)	149 (5.3)	200 (6.8)
Any Psychiatric/Mood Condition	873 (33.1)^a^	1156 (39.9)	973 (35.0)^a^	1277 (42.4)	976 (36.0)^a^	1242 (43.6)	1065 (37.8)^a^	1294 (44.1)
Delusions	158 (6.0)^a^	73 (2.5)	191 (6.9)^a^	70 (2.3)	168 (6.2)^a^	80 (2.8)	196 (7.0)^a^	85 (2.9)
Hallucinations	110 (4.2)^a^	54 (1.9)	107 (3.9)^a^	46 (1.5)	114 (4.2)^a^	59 (2.1)	116 (4.1)^a^	62 (2.1)

*Abbreviations* ADL, activities of daily living; AL, assisted living; CHESS, Changes in Health, End-Stage Disease, Signs & Symptoms; FI, frailty index; SD, standard deviation

*Notes* Unless indicated otherwise, data are expressed as Column No. (%) of residents with percentages rounded

a Standardized difference of >0.10 considered clinically meaningful difference (comparing Dementia Care to Other AL)

b Standardized difference of >0.10 considered clinically meaningful difference (comparing Dec 2021 to Mar 2019)

c Sum of 4 behaviours assessed on the RAI-HC (verbal abuse, physical abuse, socially inappropriate/disruptive care, resisting care)

The proportion of residents in urban vs. rural homes and for-profit vs. non-profit homes was comparable for the two settings (Additional File 1, Table S3). Residents in dementia care settings were more likely to reside in the Edmonton health zone and less likely in homes with the smallest bed size (i.e., 1–25).

**Fig. 1:**
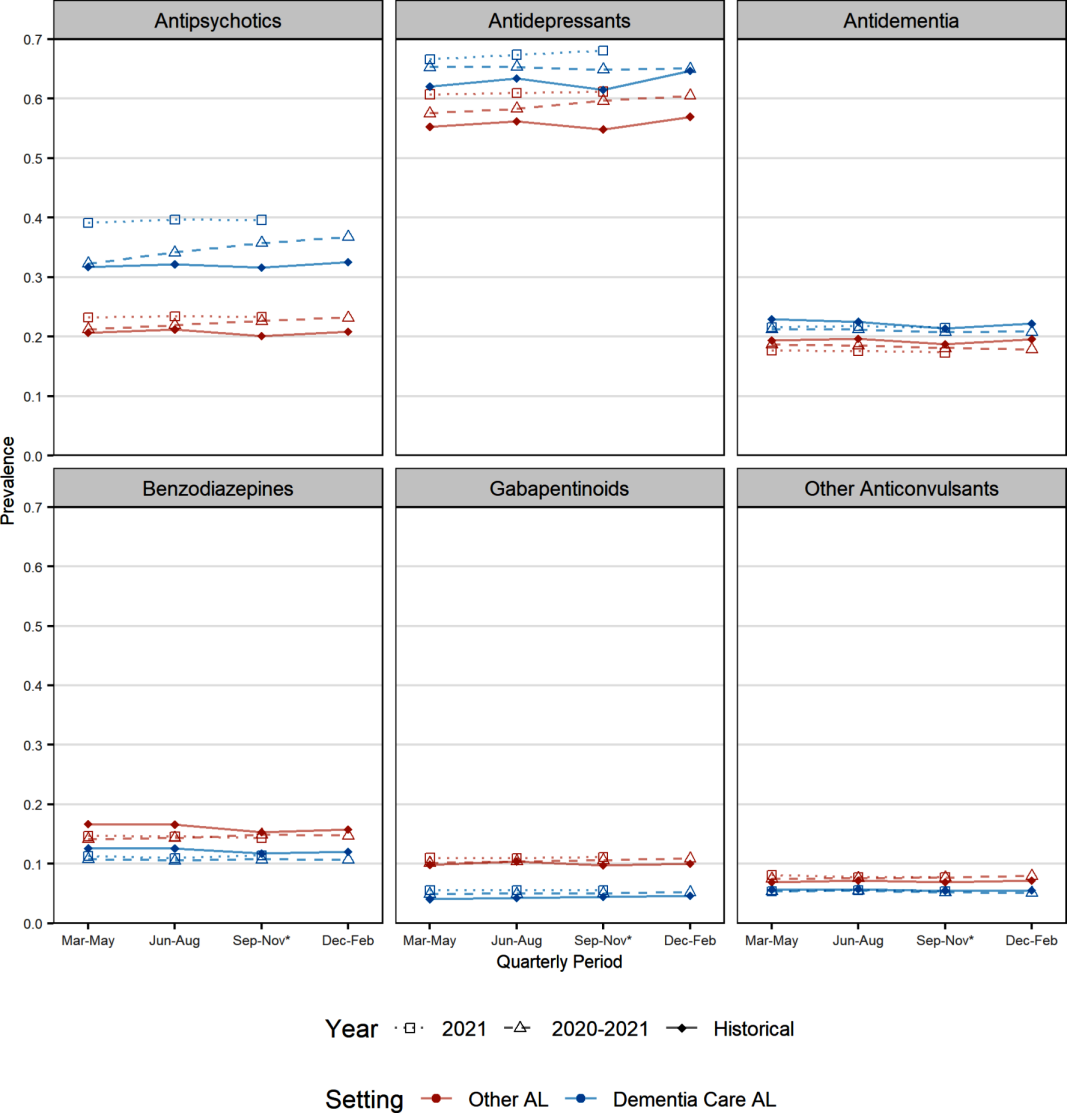
Quarterly prevalence of each CNS medication for pandemic and historical years, by setting type

Figure 1 presents the quarterly prevalence of each medication class for the historical (2018/2019 combined) and 2020–2021 pandemic periods for each AL setting (see also Additional File 1, Figure S1). There were consistent differences in the overall prevalence of each medication class between dementia care and other AL settings throughout the study period, with a higher prevalence of antipsychotic, antidepressant and anti-dementia medication use but a lower prevalence of benzodiazepines, gabapentinoids and other anticonvulsants among residents of dementia care relative to those in other AL settings. Antidepressant use was particularly common in both settings both prior to and during the pandemic period (e.g., 68% for dementia care and 61% for other AL settings by end of study). Antipsychotics persisted as the second most prevalent CNS medication class, increasing to 39.6% in dementia care and 23.3% in other AL settings by study end.

Table 2 provides the adjusted prevalence ratios (adjPR) and 95% confidence intervals (CI) for each CNS medication class associated with each pandemic quarter (vs. comparable historical periods) and AL setting (dementia care vs. other AL). In both settings, there was a statistically significant increase in antipsychotic use across pandemic waves 2–4. During waves 3 and 4, this increase was significantly greater for residents in dementia care vs. other AL settings (e.g., dementia care: adjPR 1.20, 95% CI 1.14–1.27; other AL: adjPR 1.09, 95% CI 1.02–1.17, period-setting interaction, *p* = 0.023, wave 3). For dementia care AL, the increase in antipsychotic use also occurred at an earlier period in the pandemic (prior to the start of wave 2) (see Additional File 1, Table S4 for all period & setting estimates).

**Table 2:** Adjusted prevalence ratios for each CNS medication, comparing COVID-19 pandemic vs. historical quarterly periods, by setting

	Adjusted prevalence ratio^a^ for medication use associated with select COVID-19 pandemic time periods						
**Medication class and setting**	March-May 2020 [Wave 1]	June-Aug 2020	Sept-Nov 2020 [Wave 2]	Dec 2020-Feb 2021 [Wave 2]	March – May 2021 [Wave 3]	June-Aug 2021	Sept – Dec 2021 [Wave 4]
***Antipsychotics***							
Period (pandemic vs. 2018/19)							
Dementia Care Other AL	1.02 (0.97–1.07) 1.02 (0.96–1.08)	**1.06 (1.01–1.11)** 1.02 (0.97–1.09)	**1.11 (1.06–1.17)** **1.10 (1.03–1.16)**	**1.12 (1.07–1.18)** **1.07 (1.01–1.14)**	**1.20 (1.14–1.27)** ^**b**^ **1.09 (1.02–1.17)** ^**b**^	**1.22 (1.16–1.29)** ^**c**^ **1.08 (1.01–1.16)** ^**c**^	**1.23 (1.16–1.29)** ^**d**^ **1.13 (1.05–1.21)** ^**d**^
***Antidepressants***							
Period (pandemic vs. 2018/19)							
Dementia Care Other AL	**1.05 (1.02–1.07)** **1.04 (1.01–1.07)**	1.02 (1.00-1.05) **1.04 (1.01–1.06)**	**1.05 (1.02–1.08)** **1.08 (1.06–1.11)**	1.00 (0.97–1.03)^e^ **1.05 (1.02–1.08)** ^**e**^	**1.06 (1.03–1.09)** **1.09 (1.06–1.13)**	**1.05 (1.02–1.08)** **1.09 (1.05–1.12)**	**1.09 (1.06–1.13**) **1.11 (1.08–1.15)**
***Anti-dementia***							
Period (pandemic vs. 2018/19)							
Dementia Care Other AL	0.94 (0.88–1.01) 0.97 (0.91–1.03**)**	0.95 (0.89–1.02) 0.95 (0.89–1.02)	0.99 (0.92–1.06) 0.99 (0.93–1.06)	**0.91 (0.85–0.98)** **0.90 (0.83–0.96)**	0.95 (0.87–1.02) 0.92 (0.85-1.00)	0.97 (0.90–1.05) **0.91 (0.84–0.99)**	1.02 (0.94–1.10) 0.95 (0.87–1.03)
***Benzodiazepines***							
Period (pandemic vs. 2018/19)							
Dementia Care Other AL	**0.86 (0.78–0.94)** **0.84 (0.78–0.90)**	**0.84 (0.77–0.92)** **0.86 (0.80–0.92)**	**0.91 (0.83-1.00)** 0.95 (0.88–1.02)	**0.88 (0.80–0.98)** **0.90 (0.83–0.98)**	**0.89 (0.80–0.99)** **0.86 (0.79–0.94)**	**0.87 (0.78–0.97)** **0.87 (0.80–0.95)**	0.97 (0.87–1.08) 0.93 (0.85–1.02)
***Gabapentinoids***							
Period (pandemic vs. 2018/19)							
Dementia Care Other AL	**1.21 (1.03–1.42)** 1.02 (0.92–1.12)	1.16 (0.99–1.35) 1.00 (0.91–1.10)	1.13 (0.98–1.31) 1.08 (0.98–1.19)	1.10 (0.95–1.29) 1.05 (0.95–1.17)	**1.32 (1.10–1.59)** 1.07 (0.95–1.20)	**1.23 (1.02–1.48)** 1.01 (0.90–1.14)	**1.19 (1.00-1.42)** 1.10 (0.98–1.23)
***Other Anticonvulsants***							
Period (pandemic vs. 2018/19)							
Dementia Care Other AL	0.95 (0.84–1.09) 1.08 (0.97–1.20)	0.96 (0.85–1.09) 1.03 (0.94–1.14)	0.93 (0.81–1.06) 1.04 (0.94–1.16)	0.95 (0.83–1.09) 1.08 (0.97–1.21)	0.96 (0.82–1.14) **1.15 (1.01–1.30)**	0.96 (0.82–1.12) 1.03 (0.92–1.17)	0.95 (0.81–1.11) 1.03 (0.91–1.17)

*Abbreviations* AL, assisted living; CNS, central nervous system

*Notes* Bolded estimates are statistically significant, *p*<0.05

a Models adjusted for age, sex, AL home health zone location, AL home ownership status and COVID-19 cases in AL home

b Test of statistical significance for interaction of period*setting, *p*=0.023

c Test of statistical significance for interaction of period*setting, *p*=0.005

d Test of statistical significance for interaction of period*setting, *p*=0.052

e Test of statistical significance for interaction of period*setting, *p*=0.007

Antidepressant use generally showed a modest statistically significant increase across all pandemic waves for residents of both AL settings (e.g., dementia care: adjPR 1.05, 95% CI 1.02–1.08; other AL: adjPR 1.08, 95% CI 1.06–1.11, initial months of wave 2). One exception was the absence of a statistically significant increase in prevalent antidepressant use among residents in dementia care settings during the latter months of wave 2.

For both AL settings, there was little change in the prevalence of anti-dementia medications during the pandemic periods, other than a statistically significant though small decrease evident during the latter months of wave 2.

The prevalence of benzodiazepines generally showed a statistically significant decrease during pandemic waves 1–3 for both settings (e.g., dementia care: adjPR 0.88, 95%CI 0.80–0.98; other AL: adjPR 0.90, 95% CI 0.83–0.98, latter months of wave 2).

Gabapentinoid use remained stable across pandemic periods for other AL residents but showed a statistically significant increase for residents in dementia care AL during waves 1, 3 and 4 (e.g., adjPR 1.32, 95% CI 1.10–1.59, wave 3). The use of other anticonvulsants varied little across pandemic periods for residents in both settings, other than a statistically significant small increase in prevalence during wave 3 for residents in other AL settings.

For medication classes showing an increase in prevalence throughout waves 3 and 4, this increase also persisted during the interim June-August 2021 quarter.

The above model findings remained consistent in sensitivity analyses which further adjusted for resident cognitive impairment (CPS score) (Table S5), and separately, for resident frailty (FI score) (Table S6).

The adjusted associations observed between AL resident and home characteristics and prevalent medication use for each CNS class remained consistent throughout the entire pandemic period (Additional File 1, Figure S2, Panels A-E). Specifically, females were significantly less likely than males to receive antipsychotics, but significantly more likely to receive antidepressants, anti-dementia drugs and benzodiazepines. Older age residents were significantly less likely than those aged < 75 years to receive antipsychotics, antidepressants, benzodiazepines and gabapentinoids, but significantly more likely to receive anti-dementia drugs. The presence of COVID-19 among residents in the home and ownership status were generally not associated with prevalent CNS use. There were statistically significant differences in the prevalence of CNS medications among residents in homes located in different health zones which varied by medication class.

**Fig. 2:**
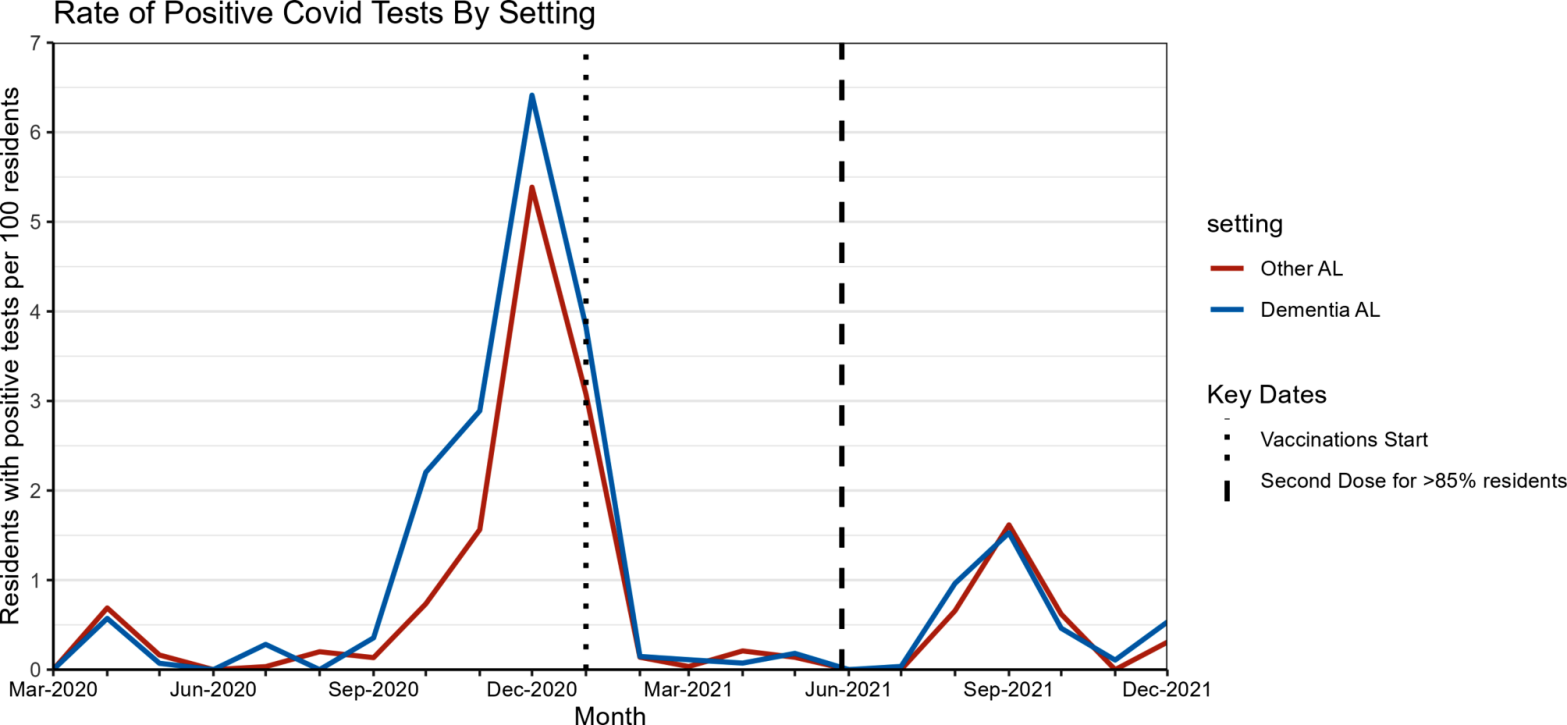
Monthly rate (per 100 residents) of positive COVID-19 tests across pandemic period, by setting type

Monthly rates of positive COVID-19 tests were generally similar in dementia care and other AL homes, except for wave 2 where positive cases emerged earlier and peaked at a higher level for residents in dementia care settings (Fig. 2, and Additional File 1, Figure S3). COVID-19 vaccination of residents in both settings started January 1, 2021 and by May 28, 2021, 85% had received their second dose.

## Discussion

This population-based study of cognitively vulnerable residents in publicly funded AL settings in Alberta showed a statistically significant increase in the prevalence of antipsychotics and antidepressants during the COVID-19 pandemic. This increase was evident for residents of both dementia care and other AL settings and persisted up to December 2021. The increase in antipsychotic use occurred earlier and was significantly more pronounced for residents in dementia care than other AL settings. Dementia care settings, but not other AL, also showed a statistically significant increase in the prevalence of gabapentinoids during several pandemic waves, though use of this medication class was relatively low throughout the study period. No meaningful changes occurred for other anticonvulsants for either setting. Anti-dementia therapy showed little variation throughout the pandemic period. Notably, benzodiazepines significantly decreased in both settings throughout much of the pandemic period.

Few investigations have explored the prevalence of CNS medications in AL settings, overall or during COVID-19, despite the increasing role of AL in caring for those at-risk for CNS drug use and related adverse events [4, 10]. Focusing on residents with dementia and/or cognitive impairment and adjusting for AL resident and home characteristics, we showed similar COVID-19 associated changes in antipsychotic, antidepressant, and benzodiazepine use to those shown in an earlier AL study that included residents with and without cognitive impairment and did not fully account for potential confounders [22]. The absolute increase in antidepressants in both settings was consistent with prior NH studies [18] while the absolute increase in antipsychotics, particularly for dementia care, was greater than previously reported for NH residents with dementia [18, 19]. Contrary to previous findings from NHs [18, 19], acute psychiatric units [37], and the general population [38] showing a statistically significant increase in benzodiazepines early in the pandemic, we found a statistically significant decline in their use in both AL settings throughout the pandemic period. Our finding may be viewed positively, particularly given the risks for poorer cognitive and health outcomes associated with benzodiazepine use in older adults [39], including poorer COVID-19 outcomes [40]. However, this may also reflect the substitution of other CNS medications (e.g., antipsychotics) for benzodiazepines in the management of responsive behaviours.

The increase in gabapentinoids and more pronounced rise in antipsychotics evident among residents in dementia care AL raises several concerns. Pre-pandemic NH research illustrated substantial increases in gabapentinoid use, often concurrent with declining trends in the use of benzodiazepines, antipsychotics, and opioids [41, 42]. Though gabapentin use may have increased in dementia care settings in response to an increase in resident pain [42, 43], these agents may have been increasingly used to treat aggression or other behaviours despite their potential risks (e.g., impaired motor function, syncope, falls) and uncertain benefits [39, 44].

Our findings are somewhat surprising as one might reasonably expect the presence of dementia-specific services and trained staff would help mitigate any increase in the potentially inappropriate use of CNS medications among this cognitively vulnerable cohort [5]. Previous NH research has shown lower rates of psychotropic medications with higher professional nurse staff hours and specific training in dementia care [45]. Higher baseline use of antipsychotics and antidepressants in dementia care (vs. other) AL residents likely reflects their higher levels of cognitive and functional impairment, depression and responsive behaviours. Yet, findings from our sensitivity analyses suggest that the more pronounced increase in prevalent antipsychotic (and gabapentinoid) use in dementia care is not fully explained by setting differences in residents’ cognitive impairment or frailty level. It is possible that dementia care AL faced greater challenges during the pandemic (e.g., with implementing infection control measures, staffing shortages, reduced access to non-pharmacologic treatments, loss of family involvement) [9, 46], leading to an increased reliance on pharmacotherapy for responsive behaviours.

The sustained higher prevalence of antipsychotic and antidepressant use among AL residents in both settings suggests this was not limited to the potentially appropriate short-term treatment of mental health distress among residents. Further, the statistically significant increase in antipsychotics, antidepressants and gabapentinoids (dementia care only) was evident during the third pandemic wave in Alberta, a period with essentially no COVID-19 cases among residents following wide-spread vaccination of the AL population. Overall, our findings support a call for further research on the drivers of persistent psychotropic drug use and health consequences for AL residents with dementia and their families [47–50].

Little information exists on whether the COVID-19 pandemic altered the use of anti-dementia medications among older persons with dementia residing in community [51] or congregate care settings [52]. Examining prescription sales data for 34 countries in Europe and North America, Ju et al. [51] showed that most countries experienced a temporary decline in anti-dementia medication sales during April and May of 2020 possibly because of disruptions to healthcare access. In a relatively small study of 252 residents with dementia in Dutch nursing homes, overall use of anti-dementia medication use was low (2–3%) and did not meaningfully change during the first pandemic wave [52]. Anti-dementia medication use was more common in our sample of AL residents (~ 20%) and showed little variation throughout the four pandemic waves except for a statistically significant, though modest, decline among residents of both settings during pandemic wave two (a period with especially high COVID-19 case rates in these settings).

Our observations of the key resident and home characteristics associated with CNS medication use during the pandemic raise additional concerns regarding pharmacotherapy in particular sub-groups of AL residents. Female residents were significantly more likely to use benzodiazepines, antidepressants, and anti-dementia therapy whereas antipsychotic use was significantly more common among male residents. The finding of generally lower psychotropic medication use among older aged residents is reassuring given the risks posed by these medications [39]. However, the substantially higher prevalence of anti-dementia therapy among residents aged 85 + years, a finding consistent with previous NH research [53], is discordant with therapeutic guidelines that caution against the use of these agents in this oldest age group because of their higher risk for adverse events [54]. Overall, the sex and age patterns in CNS medication use we observed are generally consistent with historical patterns observed for NH residents with dementia [55–57].

Neither the presence of COVID-19 among residents in the home or ownership status (for profit vs. not) were significantly associated with CNS medication use. Previous NH research has raised concerns about for-profit homes, including the underreporting of antipsychotic prescribing [58] and increased use of psychotropic and anticholinergic medications [55, 59]. The health zone of the AL home, a measure capturing community characteristics and local health system factors and policies, was significantly associated with the use of all CNS medications during the pandemic periods. Regional variation in CNS medication use among older adults has been demonstrated in previous community-based studies [60, 61], and raises interesting questions for future research on novel drivers of potentially inappropriate CNS medication use.

Strengths of this study include its: (i) utilization of population-based linked clinical and health administration data; (ii) focus on cognitively vulnerable residents of AL, an inadequately studied care setting and at-risk population; (iii) comparison of dementia care vs. other AL settings; and (iv) investigation of COVID-19 associated CNS medication trends across four pandemic waves and following adjustment for key AL resident and home characteristics. Study limitations include the absence of data on prescription indications, non-pharmacological interventions, staff COVID-19 case rates and staff characteristics (e.g., overall staffing levels, shortages, new hires, and use of redeployed and/or agency nurses), and resident health outcomes. Given these limitations, we are not able to make specific conclusions regarding the appropriateness of these medication changes. Although most resident characteristics remained relatively stable during the study period, there may have been less timely or accurate assessments available during peak pandemic periods. Lastly, our study focused exclusively on publicly subsidized AL in Alberta. Its generalizability to other regions in Canada or globally may be limited.

## Conclusions

The increased prevalence of antipsychotic and antidepressant medications in AL settings (that persisted up to and including the fourth pandemic wave) is concerning for residents with dementia and/or cognitive impairment given their vulnerability to adverse effects. The findings from this study point to an exacerbation of challenges faced by dementia care settings during the COVID-19 pandemic and concerns about pharmacotherapeutic care despite the presence of specialized dementia care staff and environments. Careful consideration of the factors underlying these CNS medications changes is required to guide policy changes and inform dementia care strategies in settings that will inevitably see an increasing proportion of residents with dementia in the coming years.

### Electronic supplementary material

Below is the link to the electronic supplementary material.

Supplementary Material 1

## Data Availability

The datasets used in the current study are held securely in coded form at the Alberta SPOR SUPPORT Unit. While legal data sharing agreements between the Alberta SPOR SUPPORT Unit and data providers (e.g., healthcare organizations and government) prohibit the Alberta SPOR SUPPORT Unit from making the datasets publicly available, access may be granted to those who meet pre-specified criteria for access, available at: https://absporu.ca/. (email: absporu@albertainnovates.ca)

## Abbreviations

ACCIS: Alberta Continuing Care Information System
ADL: Activities of Daily Living
AHS: Alberta Health Services
AL: Assisted Living
CNS: Central Nervous System
COVID-19: Coronavirus disease 2019
DAD: Discharge Abstract Database
ImmARI: Immunization & Adverse Reactions to Immunization
NH: Nursing home
PIN: Pharmaceutical Information Network
RAI-HC: Resident Assessment Instrument – Home Care
