# Supplementary material for: Associations between specialized dementia care, COVID-19 and central nervous system medication use in assisted living: a population-based repeated cross-sectional study
Source: BMC Geriatr. 2024 Aug 14;24:684. doi: 10.1186/s12877-024-05274-w (PMC11323626; doi:10.1186/s12877-024-05274-w)

**Additional File 1**

**Maxwell CJ, et al.**

***Associations between specialized dementia care, COVID-19 and central nervous system medication use in assisted living: a population-based repeated cross-sectional study***

**Table S1. Description of Alberta Provincial Clinical and Health Administrative Databases.**

**Table S2. List of Study Medications.**

**Table S3. Yearly home characteristics of AL residents with dementia and/or cognitive impairment, by setting type.**

**Table S4. Adjusted prevalence ratios for CNS medications, by COVID-19 pandemic period and AL setting.**

**Table S5. Sensitivity Analysis 1: Adjusted prevalence ratios for each CNS medication, comparing COVID-19 pandemic vs historical quarterly periods.**

**Table S6. Sensitivity Analysis 2: Adjusted prevalence ratios for each CNS medication, comparing COVID-19 pandemic vs historical quarterly periods.**

**Figure S1. Quarterly prevalence of each CNS medication across study period, by setting type.**

**Figure S2. Adjusted prevalence ratios (95% CI) for CNS medications during COVID-19 pandemic waves 1-4, associated with AL resident and home characteristics.**

**Panel A (Antipsychotics); Panel B (Antidepressants); Panel C (Anti-dementia drugs); Panel D (Benzodiazepines); Panel E (Gabapentinoids)**

**Figure S3. Rate (per 100 residents) of positive COVID-19 tests (current or past month) across pandemic period, by setting type.**

**Table S1. Description of Alberta Provincial Clinical and Health Administrative Databases.**

| **DATABASE** | **DESCRIPTION** |
| --- | --- |
| Discharge Abstract Database (DAD) | Contains patient-level data (demographic, diagnoses, procedures, relevant dates) for all admissions to acute care hospitals in Alberta.  Data available from Apr 1, 2002. |
| Pharmaceutical Information Network (PIN) Database | Contains dates, names, Drug Identification Number (DIN), Anatomical Therapeutic Chemical (ATC) codes, quantity, and days supply for all outpatient prescription medications dispensed to residents, regardless of payment source, from community pharmacies across the province, from 2008 to present.  Data available from Apr 1, 2008. |
| Vital Statistics | Contains dates of death for residents of Alberta.  Data available from Jan 1, 1999. |
| Alberta Continuing Care Information System (ACCIS) Database | Contains data for all continuing care (Designated Supportive Living [DSL] and Long-Term Care [LTC]) residents and homes in Alberta.  Includes all mandatory clinical assessments (Resident Assessment Instrument – Home Care [RAI-HC] and Resident Assessment Instrument – Minimum Data Set 2.0 [RAI-MDS 2.0) administered among DSL and LTC residents, respectively, in Alberta. These standardized assessments are performed by trained healthcare personnel and collect information on a wide range of health indices including cognitive function, performance on activities of daily living, mood and behaviours, health instability, psychosocial well-being, and clinical diagnoses.  Also available are measures of DSL/LTC home bed size, ownership status, rural/urban residence, and health zone location in the province (the latter two characteristics based on home postal code).  Data available from Jan 1, 2010 onward. |
| Provincial Laboratory COVID-19 testing data | Contains information on COVID-19 infections (year/month of diagnosis), testing for all DSL and LTC residents in Alberta. COVID-19 data available from Feb 1, 2020 onward. |
| Immunization and Adverse Reactions to Immunization (ImmARI) | Contains information on vaccines administered including date and type of vaccination. Includes vaccines administered in various settings such as public health clinics, pharmacies, and physician offices. Data were used with approval from Alberta Health (data custodian). |

**Table S2. List of Study Medications.**

| **Medication Class** | **Medications included** |
| --- | --- |
| Antipsychotics | aripiprazole, asenapine, brexpiprazole, chlorpromazine, clozapine, flupentixol, fluphenazine, haloperidol, loxapine, lurasidone, methotrimeprazine, olanzapine, paliperidone, periciazine, perphenazine, pimozide, pipotiazine, prochlorperazine, quetiapine, risperidone, trifluoperazine, ziprasidone, zuclopenthixol, |
| Antidepressants | amitriptyline, bupropion, citalopram, clomipramine, desipramine, desvenlafaxine, doxepin, duloxetine, escitalopram, fluoxetine, fluvoxamine, imipramine, mirtazapine, moclobemide, nortriptyline, paroxetine, phenelzine, sertraline, tranylcypromine, trazodone, trimipramine, tryptophan, venlafaxine, vilazodone, vortioxetine |
| Anti-dementia | donepezil, rivastigmine, galantamine, memantine |
| Benzodiazepines^*^ | alprazolam, bromazepam, chlordiazepoxide, clonazepam, clorazepate, diazepam, flurazepam, lorazepam, nitrazepam, oxazepam, temazepam, triazolam  [also included: eszopiclone, zolpidem, zopiclone] |
| Gabapentinoids | gabapentin, pregabalin |
| Other Anticonvulsants | brivaracetam, carbamazepine, clobazam, eslicarbazepine, ethosuximide, lacosamide, lamotrigine, levetiracetam, oxcarbazepine, perampanel, phenobarbital, phenytoin, primidone, topiramate, valproic acid |

^*^ Also included were the nonbenzodiazepine receptor agonists (Z-drugs): zopiclone, zolpidem, eszopiclone

**Table S3. Yearly home characteristics of AL residents with dementia and/or cognitive impairment, by setting type.**

| **Characteristic** | **March 2019** | | **March 2020** | | **March 2021** | | **Dec 2021** | |
| --- | --- | --- | --- | --- | --- | --- | --- | --- |
|  | **Dementia Care**  **(n=2641)** | **Other AL**  **(n=2899)** | **Dementia Care**  **(n=2779)** | **Other AL**  **(n=3013)** | **Dementia Care**  **(n=2711)** | **Other AL**  **(n=2851)** | **Dementia Care**  **(n=2817)** | **Other AL**  **(n=2931)** |
| Geographic Health Zone  Calgary  Central  Edmonton  North  Sound | 706 (26.7)  333 (12.6)^a^  955 (36.2)^a^  205 (7.8)  442 (16.7) | 885 (30.5)  482 (16.6)  817 (28.2)  274 (9.5)  441 (15.2) | 781 (28.1)  356 (12.8)^a^  1001 (36.0)^a^  204 (7.3)  437 (15.7) | 949 (31.5)  498 (16.5)  856 (28.4)  291 (9.7)  419 (13.9) | 740 (27.3)  387 (14.3)^a^  950 (35.0)^a^  210 (7.8)  424 (15.6) | 898 (31.5)  516 (18.1)  743 (26.1)  268 (9.4)  426 (14.9) | 776 (27.5)  412 (14.6)^a^  995 (35.3)^a^  222 (7.9)  412 (14.6) | 918 (31.3)  540 (18.4)  782 (26.7)  298 (10.2)  393 (13.4) |
| Location  Rural  Urban | 249 (9.4)  2392 (90.6) | 334 (11.5)  2565 (88.5) | 248 (8.9)  2531 (91.1) | 324 (10.8)  2689 (89.2) | 264 (9.7)  2447 (90.3) | 328 (11.5)  2523 (88.5) | 261 (9.3)  2556 (90.7) | 344 (11.7)  2587 (88.3) |
| Bed Size  1-25  26-50  51-100  101-200  >200 | 43 (1.6)^a^  565 (21.4)  1036 (39.2)^a^  706 (26.7)  291 (11.0) | 335 (11.6)  557 (19.2)  989 (34.1)  680 (23.5)  338 (11.7) | 42 (1.5)^a^  567 (20.4)  1152 (41.5)^a^  727 (26.2)  291 (10.5) | 293 (9.7)  583 (19.3)  1080 (35.8)  730 (24.2)  327 (10.9) | 51 (1.9)^a^  567 (20.9)  1121 (41.4)  701 (25.9)  271 (10.0) | 276 (9.7)  550 (19.3)  1049 (36.8)  685 (24.0)  291 (10.2) | 46 (1.6)^a^  563 (20.0)  1183 (42.0)  734 (26.1)  291 (10.3) | 241 (8.2)^b^  585 (20.0)  1102 (37.6)  693 (23.6)  310 (10.6) |
| Ownership Status  Alberta Health Services (gov’t)  Non-Profit / Voluntary  For-Profit / Private | 169 (6.4)^a^  1080 (40.9)  1392 (52.7) | 116 (4.0)  1242 (42.8)  1541 (53.2) | 158 (5.7)  1128 (40.6)  1493 (53.7) | 130 (4.3)  1280 (42.5)  1603 (53.2) | 175 (6.5)  1067 (39.4)  1469 (54.2) | 144 (5.1)  1209 (42.4)  1498 (52.5) | 178 (6.3)  1095 (38.9)  1544 (54.8) | 160 (5.5)  1179 (40.2)  1592 (54.3) |

Abbreviations: AL, assisted living; gov’t, government.

Notes: Unless indicated otherwise, data are expressed as Column No. (%) of residents with percentages rounded

a Standardized difference of >0.10 considered clinically meaningful difference (comparing Dementia Care to Other AL)

b Standardized difference of >0.10 considered clinically meaningful difference (comparing Dec 2021 to Mar 2019)

**Table S4. Adjusted prevalence ratios for CNS medications, by COVID-19 pandemic period and AL setting.**

|  | **Adjusted Prevalence Ratio^a^ for Medication Use Associated with Select COVID-19 Pandemic Time Periods** | | | | | | |
| --- | --- | --- | --- | --- | --- | --- | --- |
| **Medication Class and Period/Setting Estimates** | March-May 2020  [Wave 1] | June-Aug 2020 | Sept-Nov 2020  [Wave 2] | Dec 2020-Feb 2021 [Wave 2] | March – May 2021 [Wave 3] | June-Aug 2021 | Sept – Dec 2021  [Wave 4] |
| ***Antipsychotics*** |  |  |  |  |  |  |  |
| Period (pandemic vs 2018/19)  Dementia Care  Other AL | 1.02 (0.97-1.07)  1.02 (0.96-1.08) | **1.06 (1.01-1.11)**  1.02 (0.97-1.09) | **1.11 (1.06-1.17)**  **1.10 (1.03-1.16)** | **1.12 (1.07-1.18)**  **1.07 (1.01-1.14)** | **1.20 (1.14-1.27)^b^**  **1.09 (1.02-1.17)^b^** | **1.22 (1.16-1.29)^c^**  **1.08 (1.01-1.16)^c^** | **1.23 (1.16-1.29)^d^**  **1.13 (1.05-1.21)^d^** |
| Setting (Dem vs Other AL)  Pandemic  2018/19 | **1.47 (1.35-1.59)**  **1.46 (1.36-1.58)** | **1.51 (1.39-1.63)**  **1.45 (1.35-1.57)** | **1.52 (1.41-1.65)**  **1.51 (1.40-1.62)** | **1.55 (1.44-1.68)**  **1.48 (1.38-1.59)** | **1.62 (1.51-1.75)**  **1.47 (1.36-1.58)** | **1.64 (1.52-1.77)**  **1.45 (1.35-1.56)** | **1.63 (1.52-1.76)**  **1.50 (1.40-1.62)** |
| ***Antidepressants*** |  |  |  |  |  |  |  |
| Period (pandemic vs 2018/19)  Dementia Care  Other AL | **1.05 (1.02-1.07)**  **1.04 (1.01-1.07)** | 1.02 (1.00-1.05)  **1.04 (1.01-1.06)** | **1.05 (1.02-1.08)**  **1.08 (1.06-1.11)** | 1.00 (0.97-1.03)^e^  **1.05 (1.02-1.08)^e^** | **1.06 (1.03-1.09)**  **1.09 (1.06-1.13)** | **1.05 (1.02-1.08)**  **1.09 (1.05-1.12)** | **1.09 (1.06-1.13**)  **1.11 (1.08-1.15)** |
| Setting (Dem vs Other AL)  Pandemic  2018/19 | **1.10 (1.06-1.15)**  **1.10 (1.05-1.14)** | **1.09 (1.05-1.14)**  **1.10 (1.06-1.15)** | **1.06 (1.02-1.10)**  **1.10 (1.06-1.14)** | **1.05 (1.01-1.09)**  **1.10 (1.06-1.14)** | **1.06 (1.02-1.11)**  **1.10 (1.06-1.14)** | **1.07 (1.03-1.11)**  **1.10 (1.06-1.15)** | **1.08 (1.04-1.12)**  **1.09 (1.06-1.13)** |
| ***Anti-dementia*** |  |  |  |  |  |  |  |
| Period (pandemic vs 2018/19)  Dementia Care  Other AL | 0.94 (0.88-1.01)  0.97 (0.91-1.03**)** | 0.95 (0.89-1.02)  0.95 (0.89-1.02) | 0.99 (0.92-1.06)  0.99 (0.93-1.06) | **0.91 (0.85-0.98)**  **0.90 (0.83-0.96)** | 0.95 (0.87-1.02)  0.92 (0.85-1.00) | 0.97 (0.90-1.05)  **0.91 (0.84-0.99)** | 1.02 (0.94-1.10)  0.95 (0.87-1.03) |
| Setting (Dem vs Other AL)  Pandemic  2018/19 | 1.08 (0.98-1.20)  **1.11 (1.02-1.22)** | 1.08 (0.98-1.19)  1.08 (0.99-1.18) | 1.09 (0.98-1.20)  1.09 (1.00-1.19) | 1.09 (0.99-1.21)  1.07 (0.98-1.17) | **1.15 (1.03-1.27)**  **1.12 (1.02-1.22)** | **1.16 (1.04-1.28)**  1.08 (0.99-1.19) | **1.17 (1.05-1.30)**  1.09 (0.99-1.19) |
| ***Benzodiazepines*** |  |  |  |  |  |  |  |
| Period (pandemic vs 2018/19)  Dementia Care  Other AL | **0.86 (0.78-0.94)**  **0.84 (0.78-0.90)** | **0.84 (0.77-0.92)**  **0.86 (0.80-0.92)** | **0.91 (0.83-1.00)**  0.95 (0.88-1.02) | **0.88 (0.80-0.98)**  **0.90 (0.83-0.98)** | **0.89 (0.80-0.99)**  **0.86 (0.79-0.94)** | **0.87 (0.78-0.97)**  **0.87 (0.80-0.95)** | 0.97 (0.87-1.08)  0.93 (0.85-1.02) |
| Setting (Dem vs Other AL)  Pandemic  2018/19 | **0.75 (0.66-0.86)**  **0.74 (0.66-0.82)** | **0.73 (0.64-0.83)**  **0.74 (0.66-0.82)** | **0.72 (0.63-0.82)**  **0.75 (0.67-0.84)** | **0.73 (0.64-0.83)**  **0.75 (0.67-0.83)** | **0.76 (0.67-0.87)**  **0.74 (0.66-0.82)** | **0.74 (0.65-0.84)**  **0.74 (0.66-0.82)** | **0.78 (0.69-0.89)**  **0.75 (0.67-0.84)** |
| ***Gabapentinoids*** |  |  |  |  |  |  |  |
| Period (pandemic vs 2018/19)  Dementia Care  Other AL | **1.21 (1.03-1.42)**  1.02 (0.92-1.12) | 1.16 (0.99-1.35)  1.00 (0.91-1.10) | 1.13 (0.98-1.31)  1.08 (0.98-1.19) | 1.10 (0.95-1.29)  1.05 (0.95-1.17) | **1.32 (1.10-1.59)**  1.07 (0.95-1.20) | **1.23 (1.02-1.48)**  1.01 (0.90-1.14) | **1.19 (1.00-1.42)**  1.10 (0.98-1.23) |
| Setting (Dem vs Other AL)  Pandemic  2018/19 | **0.48 (0.40-0.58)**  **0.41 (0.34-0.49)** | **0.47 (0.39-0.57)**  **0.41 (0.34-0.49)** | **0.47 (0.39-0.57)**  **0.45 (0.38-0.54)** | **0.48 (0.40-0.58)**  **0.46 (0.39-0.55)** | **0.50 (0.42-0.60)**  **0.40 (0.34-0.49)** | **0.50 (0.41-0.60)**  **0.41 (0.34-0.49)** | **0.49 (0.41-0.59)**  **0.45 (0.38-0.54)** |
| ***Other Anticonvulsants*** |  |  |  |  |  |  |  |
| Period (pandemic vs 2018/19)  Dementia Care  Other AL | 0.95 (0.84-1.09)  1.08 (0.97-1.20) | 0.96 (0.85-1.09)  1.03 (0.94-1.14) | 0.93 (0.81-1.06)  1.04 (0.94-1.16) | 0.95 (0.83-1.09)  1.08 (0.97-1.21) | 0.96 (0.82-1.14)  **1.15 (1.01-1.30)** | 0.96 (0.82-1.12)  1.03 (0.92-1.17) | 0.95 (0.81-1.11)  1.03 (0.91-1.17) |
| Setting (Dem vs Other AL)  Pandemic  2018/19 | **0.72 (0.59-0.87)**  **0.81 (0.68-0.97)** | **0.73 (0.61-0.88)**  **0.79 (0.66-0.94)** | **0.69 (0.57-0.84)**  **0.77 (0.65-0.92)** | **0.66 (0.55-0.80)**  **0.75 (0.63-0.90)** | **0.68 (0.56-0.82)**  **0.81 (0.68-0.97)** | **0.73 (0.61-0.88)**  **0.79 (0.66-0.94)** | **0.71 (0.58-0.85)**  **0.77 (0.64-0.92)** |

Abbreviations: AL, assisted living; CNS, central nervous system; Dem, Dementia

Notes: Bolded estimates are statistically significant, p<0.05

a Models adjusted for age, sex, AL home health zone location, AL home ownership status and COVID-19 cases in AL home

b test of statistical significance for interaction of period*setting, p=0.023

c test of statistical significance for interaction of period*setting, p=0.005

d test of statistical significance for interaction of period*setting, p=0.052

e test of statistical significance for interaction of period*setting, p=0.007

**Table S5. Sensitivity Analysis 1: Adjusted prevalence ratios for each CNS medication, comparing COVID-19 pandemic vs historical quarterly periods, by setting.**

|  | **Adjusted Prevalence Ratio^a^ for Medication Use Associated with Select COVID-19 Pandemic Time Periods** | | | | | | |
| --- | --- | --- | --- | --- | --- | --- | --- |
| **Medication Class and Setting** | March-May 2020  [Wave 1] | June-Aug 2020 | Sept-Nov 2020  [Wave 2] | Dec 2020-Feb 2021 [Wave 2] | March – May 2021 [Wave 3] | June-Aug 2021 | Sept – Dec 2021  [Wave 4] |
| ***Antipsychotics*** |  |  |  |  |  |  |  |
| Period (pandemic vs 2018/19)  Dementia Care  Other AL | 1.02 (0.97-1.07)  1.02 (0.96-1.08) | **1.06 (1.01-1.11)**  1.03 (0.97-1.09) | **1.11 (1.06-1.17)**  **1.10 (1.03-1.16)** | **1.13 (1.07-1.18)**  **1.07 (1.01-1.14)** | **1.21 (1.14-1.27)^b^**  **1.09 (1.02-1.17)^b^** | **1.23 (1.16-1.29)^c^**  **1.08 (1.01-1.16)^c^** | **1.23 (1.17-1.30)^d^**  **1.13 (1.05-1.21)^d^** |
| CPS 3+ (vs <3) | **1.10 (1.03-1.17)** | 1.07 (1.00-1.14) | 1.05 (0.99-1.12) | 1.04 (0.98-1.11) | **1.07 (1.00-1.13)** | **1.06 (1.00-1.13)** | **1.07 (1.01-1.13)** |
| ***Antidepressants*** |  |  |  |  |  |  |  |
| Period (pandemic vs 2018/19)  Dementia Care  Other AL | **1.05 (1.02-1.07)**  **1.04 (1.01-1.07)** | 1.02 (1.00-1.05)  **1.04 (1.01-1.06)** | **1.05 (1.02-1.08)**  **1.08 (1.06-1.11)** | 1.00 (0.97-1.03)^e^  **1.05 (1.02-1.08)^e^** | **1.06 (1.03-1.09)**  **1.09 (1.06-1.13)** | **1.05 (1.02-1.08)**  **1.09 (1.05-1.12)** | **1.09 (1.06-1.13**)  **1.11 (1.08-1.15)** |
| CPS 3+ (vs <3) | 1.00 (0.97-1.04) | 1.00 (0.97-1.03) | 0.99 (0.95-1.02) | 1.00 (0.96-1.03) | 0.99 (0.96-1.02) | 0.98 (0.95-1.01) | 0.99 (0.96-1.02) |
| ***Anti-dementia*** |  |  |  |  |  |  |  |
| Period (pandemic vs 2018/19)  Dementia Care  Other AL | **0.93 (0.87-1.00)**  0.96 (0.90-1.02**)** | 0.94 (0.88-1.00)  0.94 (0.88-1.01) | 0.98 (0.91-1.05)  0.99 (0.92-1.05) | **0.91 (0.84-0.98)**  **0.89 (0.83-0.96)** | 0.93 (0.86-1.01)  **0.91 (0.84-0.99)** | 0.96 (0.88-1.04)  **0.90 (0.83-0.98)** | 1.01 (0.93-1.09)  0.94 (0.87-1.02) |
| CPS 3+ (vs <3) | **0.73 (0.67-0.79)** | **0.74 (0.68-0.80)** | **0.76 (0.70-0.83)** | **0.77 (0.71-0.84)** | **0.74 (0.68-0.80)** | **0.74 (0.69-0.80)** | **0.76 (0.70-0.82)** |
| ***Benzodiazepines*** |  |  |  |  |  |  |  |
| Period (pandemic vs 2018/19)  Dementia Care  Other AL | **0.86 (0.78-0.94)**  **0.84 (0.78-0.90)** | **0.84 (0.77-0.92)**  **0.85 (0.79-0.92)** | **0.91 (0.83-1.00)**  0.95 (0.88-1.02) | **0.88 (0.80-0.98)**  **0.90 (0.83-0.98)** | **0.89 (0.80-0.99)**  **0.86 (0.78-0.93)** | **0.87 (0.78-0.96)**  **0.86 (0.79-0.94)** | 0.97 (0.87-1.07)  0.93 (0.85-1.02) |
| CPS 3+ (vs <3) | 0.95 (0.86-1.06) | 0.94 (0.85-1.04) | 0.94 (0.85-1.04) | 0.93 (0.84-1.03) | **0.91 (0.82-1.00)** | 0.91 (0.83-1.00) | 0.91 (0.83-1.00) |
| ***Gabapentinoids*** |  |  |  |  |  |  |  |
| Period (pandemic vs 2018/19)  Dementia Care  Other AL | **1.20 (1.03-1.41)**  1.01 (0.91-1.12) | 1.15 (0.99-1.35)  1.00 (0.91-1.10) | 1.13 (0.98-1.31)  1.08 (0.98-1.19) | 1.10 (0.95-1.28)  1.05 (0.95-1.17) | **1.31 (1.09-1.57)^f^**  1.06 (0.94-1.19)^f^ | **1.22 (1.02-1.47)**  1.01 (0.90-1.13) | **1.19 (1.00-1.41)**  1.09 (0.97-1.22) |
| CPS 3+ (vs <3) | **0.77 (0.66-0.90)** | **0.78 (0.67-0.91)** | **0.79 (0.68-0.92)** | **0.81 (0.70-0.94)** | **0.79 (0.69-0.91)** | **0.80 (0.69-0.91)** | **0.79 (0.69-0.90)** |

Abbreviations: AL, assisted living; CNS, central nervous system; CPS, cognitive performance scale

Notes: Bolded estimates are statistically significant, p<0.05

a Models adjusted for age, sex, cognitive performance score (CPS 3+ vs <3), AL home health zone location, AL home ownership status and COVID-19 cases in AL home

b Test of statistical significance for interaction of period*setting, p=0.023

c Test of statistical significance for interaction of period*setting, p=0.004

d Test of statistical significance for interaction of period*setting, p=0.043

e Test of statistical significance for interaction of period*setting, p=0.007

f Test of statistical significance for interaction of period*setting, p=0.058

**Table S6. Sensitivity Analysis 2: Adjusted prevalence ratios for each CNS medication, comparing COVID-19 pandemic vs historical quarterly periods, by setting.**

|  | **Adjusted Prevalence Ratio^a^ for Medication Use Associated with Select COVID-19 Pandemic Time Periods** | | | | | | |
| --- | --- | --- | --- | --- | --- | --- | --- |
| **Medication Class and Setting** | March-May 2020  [Wave 1] | June-Aug 2020 | Sept-Nov 2020  [Wave 2] | Dec 2020-Feb 2021 [Wave 2] | March – May 2021 [Wave 3] | June-Aug 2021 | Sept – Dec 2021  [Wave 4] |
| ***Antipsychotics*** |  |  |  |  |  |  |  |
| Period (pandemic vs 2018/19)  Dementia Care  Other AL | 1.02 (0.97-1.07)  1.02 (0.96-1.08) | **1.06 (1.01-1.11)**  1.02 (0.97-1.09) | **1.11 (1.06-1.16)**  **1.10 (1.03-1.16)** | **1.12 (1.07-1.18)**  **1.07 (1.01-1.14)** | **1.20 (1.14-1.27)^b^**  **1.09 (1.01-1.16)^b^** | **1.23 (1.16-1.29)^c^**  **1.08 (1.01-1.16)^c^** | **1.23 (1.17-1.30)^d^**  **1.13 (1.05-1.21)^d^** |
| FI >0.3 (vs ≤ 0.3) | **1.12 (1.05-1.19)** | **1.10 (1.04-1.17)** | **1.12 (1.05-1.18)** | **1.11 (1.05-1.18)** | **1.12 (1.06-1.18)** | **1.12 (1.06-1.18)** | **1.14 (1.08-1.20)** |
| ***Antidepressants*** |  |  |  |  |  |  |  |
| Period (pandemic vs 2018/19)  Dementia Care  Other AL | **1.05 (1.02-1.07)**  **1.04 (1.01-1.07)** | 1.02 (1.00-1.05)  **1.04 (1.01-1.06)** | **1.05 (1.02-1.07)**  **1.08 (1.05-1.11)** | 1.00 (0.97-1.02)^e^  **1.05 (1.02-1.08)^e^** | **1.06 (1.03-1.09)**  **1.09 (1.06-1.13)** | **1.05 (1.02-1.08)**  **1.08 (1.05-1.12)** | **1.09 (1.06-1.12**)  **1.11 (1.08-1.15)** |
| FI >0.3 (vs ≤ 0.3) | **1.12 (1.09-1.16)** | **1.10 (1.07-1.14)** | **1.11 (1.07-1.14)** | **1.11 (1.08-1.14)** | **1.11 (1.08-1.14)** | **1.09 (1.06-1.13)** | **1.11 (1.08-1.14)** |
| ***Anti-dementia*** |  |  |  |  |  |  |  |
| Period (pandemic vs 2018/19)  Dementia Care  Other AL | **0.94 (0.88-1.00)**  0.97 (0.91-1.03**)** | 0.94 (0.89-1.01)  0.95 (0.89-1.01) | 0.99 (0.92-1.06)  0.99 (0.93-1.06) | **0.91 (0.85-0.98)**  **0.90 (0.83-0.96)** | 0.94 (0.87-1.02)  0.92 (0.85-1.00) | 0.96 (0.89-1.04)  **0.91 (0.84-0.99)** | 1.01 (0.94-1.10)  0.94 (0.87-1.02) |
| FI >0.3 (vs ≤ 0.3) | **0.66 (0.60-0.72)** | **0.66 (0.60-0.72)** | **0.66 (0.60-0.72)** | **0.67 (0.62-0.73)** | **0.67 (0.62-0.73)** | **0.66 (0.61-0.71)** | **0.67 (0.62-0.73)** |
| ***Benzodiazepines*** |  |  |  |  |  |  |  |
| Period (pandemic vs 2018/19)  Dementia Care  Other AL | **0.86 (0.78-0.94)**  **0.84 (0.78-0.90)** | **0.84 (0.77-0.93)**  **0.86 (0.80-0.92)** | **0.91 (0.83-1.00)**  0.95 (0.88-1.02) | **0.88 (0.80-0.98)**  **0.90 (0.83-0.98)** | **0.89 (0.80-0.99)**  **0.86 (0.79-0.94)** | **0.87 (0.78-0.97)**  **0.87 (0.79-0.94)** | 0.97 (0.87-1.08)  0.93 (0.85-1.02) |
| FI >0.3 (vs ≤ 0.3) | 1.08 (0.99-1.19) | 1.06 (0.96-1.17) | 1.08 (0.98-1.18) | 1.08 (0.98-1.19) | 1.08 (0.98-1.18) | 1.07 (0.98-1.17) | 1.05 (0.96-1.15) |
| ***Gabapentinoids*** |  |  |  |  |  |  |  |
| Period (pandemic vs 2018/19)  Dementia Care  Other AL | **1.22 (1.04-1.43)**  1.02 (0.92-1.12) | 1.16 (1.00-1.36)  1.00 (0.91-1.10) | 1.13 (0.98-1.31)  1.08 (0.98-1.19) | 1.10 (0.95-1.29)  1.05 (0.95-1.17) | **1.33 (1.10-1.60)^f^**  1.06 (0.95-1.20)^f^ | **1.23 (1.03-1.48)**  1.01 (0.90-1.13) | **1.20 (1.00-1.42)**  1.10 (0.98-1.23) |
| FI >0.3 (vs ≤ 0.3) | **1.28 (1.11-1.48)** | **1.32 (1.15-1.52)** | **1.28 (1.12-1.47)** | **1.28 (1.12-1.47)** | **1.34 (1.17-1.53)** | **1.37 (1.20-1.56)** | **1.28 (1.13-1.45)** |

Abbreviations: AL, assisted living; CNS, central nervous system; FI, frailty index

Notes: Bolded estimates are statistically significant, p<0.05

a Models adjusted for age, sex, frailty (FI >0.3 vs ≤ 0.3), AL home health zone location, AL home ownership status and COVID-19 cases in AL home

b Test of statistical significance for interaction of period*setting, p=0.022

c Test of statistical significance for interaction of period*setting, p=0.004

d Test of statistical significance for interaction of period*setting, p=0.052

e Test of statistical significance for interaction of period*setting, p=0.005

f Test of statistical significance for interaction of period*setting, p=0.048

**Figure S1. Quarterly prevalence of each CNS medication across study period, by setting type.**


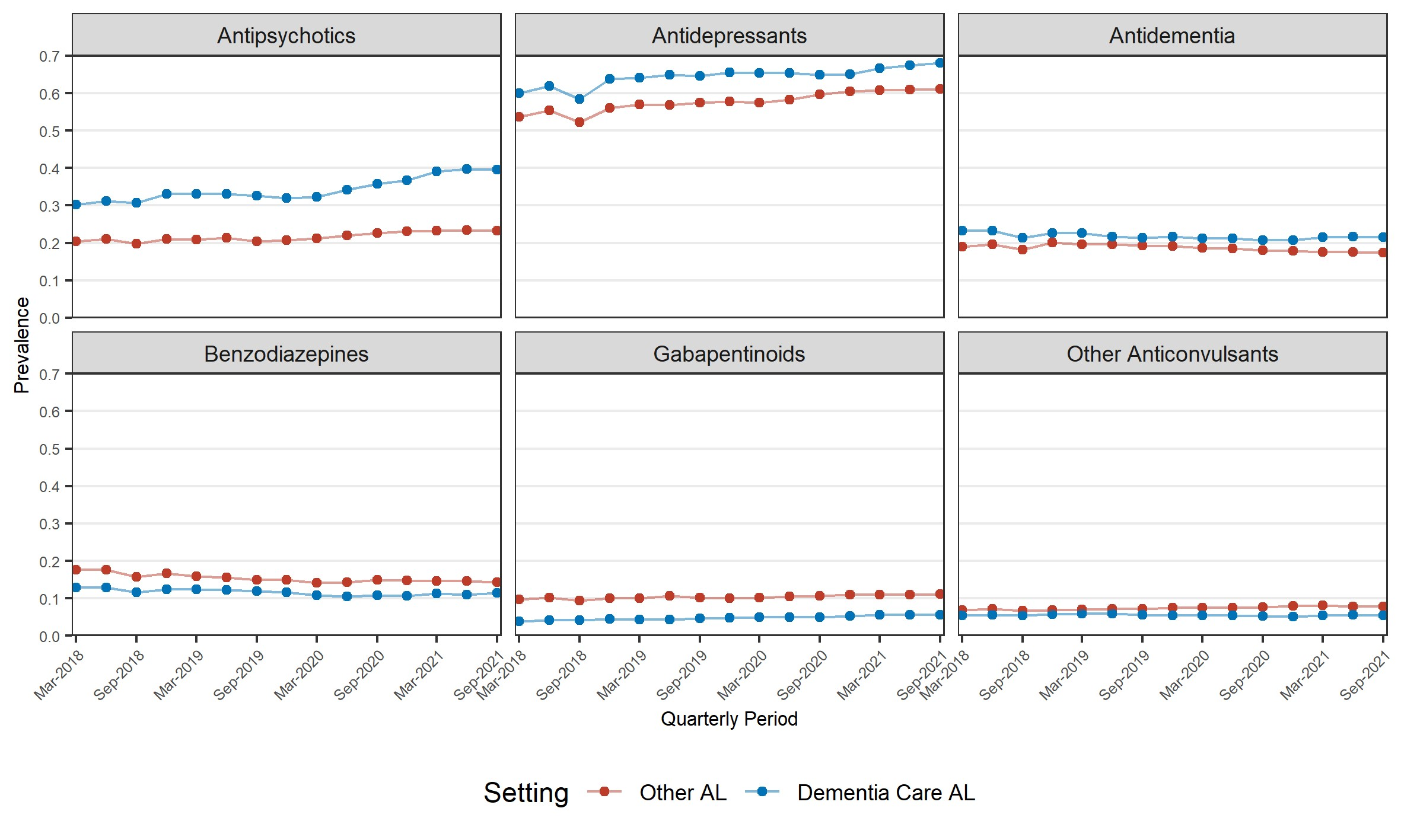


**Figure S2. Adjusted prevalence ratios (95%CI) for CNS medications during COVID-19 pandemic waves 1-4, associated with AL resident and home characteristics.**

**Panel A (Antipsychotics)**

**Panel B (Antidepressants)**

**Panel C (Anti-dementia drugs)**

**Panel D (Benzodiazepines)**

**Panel E (Gabapentinoids)**

**Figure S3. Rate (per 100 residents) of positive COVID-19 tests (current or past month) across pandemic period, by setting type.**


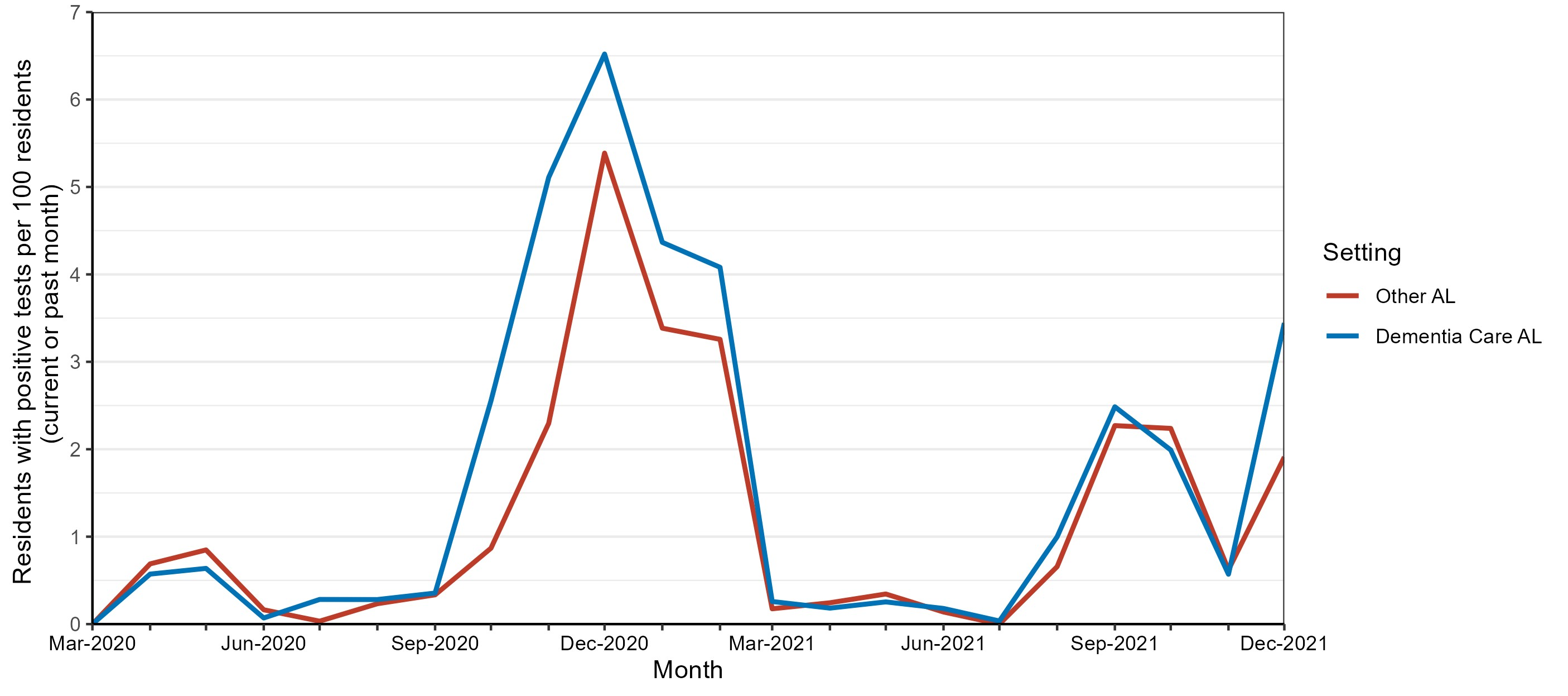

Supplement: Supplementary file 1 — Supplementary Material 1 [file 12877_2024_5274_MOESM1_ESM.docx]
